# A survey of free-ranging deer in Ireland for serological evidence of exposure to bovine viral diarrhoea virus, bovine herpes virus-1, bluetongue virus and Schmallenberg virus

**DOI:** 10.1186/s13620-017-0091-z

**Published:** 2017-05-12

**Authors:** David A. Graham, Clare Gallagher, Ruth F. Carden, Jose-Maria Lozano, John Moriarty, Ronan O’Neill

**Affiliations:** 1 Animal Health Ireland, 4-5 The Archways, Carrick on Shannon, Co. Leitrim Ireland; 2 Central Veterinary Research Laboratory, Backweston Campus, Celbridge, Ireland; 3 Adjunct Research Fellow, School of Archaeology, University College Dublin, Belfield, Dublin 4 Ireland

**Keywords:** Deer, Ireland, Serology, bovine viral diarrhoea virus, bovine herpesvirus-1, Schmallenberg virus, Bluetongue virus, Survey

## Abstract

**Background:**

Deer are an important wildlife species in both the Republic of Ireland and Northern Ireland having colonised most regions across the island of Ireland. In comparison to cattle and sheep which represent the main farmed ruminant species on the island, there is a lack of data concerning their exposure, as measured by the presence of antibodies, to important viral pathogens of ruminants. A study was therefore undertaken to investigate the seroprevalence of wild deer to four viruses, namely bovine viral diarrhoea virus (BVDV), bovine herpesvirus-1 (BoHV-1), Schmallenberg virus (SBV) and bluetongue virus (BTV).

**Results:**

Two panels of sera were assembled; Panel 1 comprised 259 samples (202 collected in the Republic of Ireland and 57 in Northern Ireland) between 2013 and 2015, while Panel 2 comprised 131 samples collected in the Republic of Ireland between 2014 and 2015. Overall sika deer (*Cervus nippon*) were sampled most commonly (54.8%), followed by fallow deer (*Dama dama*) (35.3%), with red deer (*Cervus elaphus*) (4.3%) and hybrid species (0.3%) sampled less frequently, with the species not being recorded for the remaining 5.3% of deer sampled. Age was not recorded for 96 of the 390 deer sampled. 196 of the remainder were adults, while 68 and 30 were yearlings and calves, respectively. Using commercially available enzyme-linked immunosorbent assays, true prevalence and 95% confidence intervals were calculated as 9.9%, (6.8-13.0% CI), SBV; 1.5% (0.1-3.0% CI), BoHV-1; 0.0%, 0-1.7% CI), BVDV; and 0.0%, (0.01-0.10% CI), BTV.

**Conclusions:**

The results indicate a very low seroprevalence for both BVDV and BoHV-1 in the wild deer tested within the study and, are consistent with a very low prevalence in Ireland. While serological cross-reaction with cervid herpesviruses cannot be excluded, the results in both cases suggest that the presence of these viruses in deer is not a significant risk to their control and eradication from the cattle population. This is important given the ongoing programme to eradicate BVDV in Ireland and deliberations on a national eradication programme for BoHV-1. The SBV results show consistency with those reported from cattle and sheep on the island of Ireland, while the BTV results are consistent with this virus remaining exotic to Ireland. The results provide a baseline against which future surveys of either wild or farmed/captive deer populations can be compared.

## Background

Deer are the largest terrestrial wild mammal and an important wildlife species on the island of Ireland, with species including fallow (*Dama dama*), sika (*Cervus nippon*) and red deer (*Cervus elaphus*) present. While the population of red deer in Killarney, Co. Kerry are descendants of a c.5,000 year old early introduction to the island [1], other populations of red and sika deer date back for only approximately 150 years, whilst muntjac deer and roe deer are relatively recent introductions although the abundance and distribution range of these two species is relatively unknown [2]. The presence of muntjac deer (*Muntiacus* sp) was confirmed in Northern Ireland in 2009 and 2015. This species has also been reported previously in the Republic of Ireland but there have been no verified sightings since March 2009 [3]. The presence of roe deer (*Capreolus capreolus*) in Northern Ireland was verified for the first time in 2014 [4]. In common with other European countries, the ranges of red, fallow and sika deer in Ireland has increased markedly over the past 30 years, with compound annual rates of expansion of 3–7% depending on species [2]. This expansion has created a number of concerns, including potential detrimental ecological impacts, damage to protected environments, conflict with commercial land use objectives, collisions with vehicles and increased risk of disease transmission, both among deer and between deer and other species [2].

As ruminants, deer share susceptibility to a number of infectious diseases that affect farmed ruminants including cattle, sheep and goats. The dynamics of interactions between these populations can be complex. Depending on the particular pathogen and ecological factors, deer populations in Ireland could potentially maintain a pathogen through a sylvatic cycle independent of farmed populations. They could also acquire infection from farmed populations with which they come into contact. This could result in spillover, where the infection does not become established, or conversely the establishment of deer as reservoir (maintenance) hosts from which infection could be transmitted back to farmed ruminants. Finally, for vector-borne pathogens such as blue tongue virus (BTV) and Schmallenberg virus (SBV) that lack mammalian host specificity [5, 6], deer and farmed animals could both be part of wider transmission pathways involving vector species. Little is currently known about the prevalence or distribution in Ireland of a range of infectious diseases that have the potential to affect wild deer populations. The potential for deer to act as a reservoir for a number of viral diseases of farmed ruminants has recently become of concern in Ireland. This follows the introduction of a national eradication programme for bovine viral diarrhoea virus (BVDV) in cattle [7, 8], discussions about a possible national eradication programme for bovine herpes virus-1 (BoHV-1), and the emergence in Europe of blue tongue virus (BTV) and Schmallenberg virus (SBV).

BVDV, a member of the genus *Pestivirus*, family *Flaviviridae* [9], can cause a range of reproductive problems including abortion, mummification and congenital birth defects, with the birth of persistently infected (PI) offspring as a consequence of *in utero* infection in early pregnancy [9]. These PI animals are key to the epidemiology of the disease, and their identification and removal is a central part of eradication programmes [10]. Historically, infection with BVDV has been widespread in cattle in both the Republic of Ireland (ROI) and Northern Ireland (NI) [11, 12].

While primarily a pathogen of cattle, BVDV can infect a wide range of other domestic and wild species, including sheep, goats, pigs, camelids, and red, fallow, sika, roe and white-tail (*Odocoileus virgianianus*) deer [13], with the generation of PI does described previously under experimental conditions following inoculation of pregnant females with BVDV or co-habitation with PI cattle [14, 15]. Evidence of infection of sheep in Ireland has been published previously, [16, 17], with lower levels of exposure in sheep reported relative to cattle. While antibodies to BVDV have been detected previously in deer in Ireland (unpublished data), there are no published prevalence studies.

BoHV-1 is a member of the genus *Varicellovirus*, sub-family *Alphaherpesvirinae*, family *Herpesviridae* [18]. It is the aetiological agent for infectious bovine rhinotracheitis (IBR), with abortion and milk drop also being sequelae to infection [19]. It emerged as a significant cause of outbreaks of respiratory disease in ROI in the 1990s and is now widespread in cattle herds across the whole island of Ireland [12, 20, 21].

There is evidence from experimental studies that deer are susceptible to infection with BoHV-1 [22, 23] and the presence of the virus has been confirmed by PCR and sequencing in free-ranging red, roe and fallow deer [24]. Other alphaherpesviruses of the *Varicellovirus* genus, closely related to BoHV-1, have been isolated from deer, including cervid herpesvirus-1 (CvHV-1) and cervid herpesvirus-2 (CvHV-2). CvHV-1 is recognised as the cause of an ocular syndrome in red deer and has also been associated with lesions in the reproductive tract similar to those seen in cattle with infectious pustular vulvo-vaginitis due to BoHV-1, whereas CvHV-2 results in a sub-clinical genital infection [25, 26]. There is a high degree of serological cross-reactivity between BoHV-1 and CvHV-1 and -2 [23, 25, 27, 28]. Experimental studies have demonstrated that cattle can be infected with CvHV-1 and -2 [25].

SBV is a member of the Simbu serogroup within the genus *Orthobunyavirus*, family *Bunyaviridae.* In common with other members of the Simbu serogroup, SBV is arthropod-borne, relying for transmission on insect vectors, of which biting midges *Culicoides spp* are considered the most important. SBV is not species-specific, being capable of infecting a wide range of ruminants, including deer. The most important outcome of infection is the induction of severe congenital defects in newborn calves, lambs and kids, premature births and the birth of stillborn or mummified foetuses, resulting from infection at a critical stage of pregnancy [5].

In contrast to BVDV and BoHV-1 which are well established in cattle in Ireland and Europe, disease associated with SBV was described for the first time in Germany in 2011, with no evidence for the presence of the virus in Europe prior to then [5]. The first cases in both ROI and NI were reported in cattle in October 2012 [29, 30]. Infection in ROI is believed to have been introduced in the summer of 2012 in the southeast of the country, with subsequent spread outwards, although evidence suggests that the anticipated nationwide spread in 2013 stalled [31–34].

Bluetongue is a disease of ruminants, including deer, caused by bluetongue virus (BTV), a member of the genus *Orbivirus*, family *Reoviridae* [6, 35], of which 24 different serotypes have been identified to date. Infection with BTV can produce a wide range of clinical signs, particularly in sheep. Alternatively, infection may be sub-clinical. In common with SBV, the primary means of transmission between ruminant hosts is through a number of species of biting midges of the genus *Culicoides*. Other transmission routes, including transplacentally, via direct contact and through semen have also been identified [6, 36].

Historically, BTV has been a disease of Africa and the Middle East, with occasional outbreaks in Southern Europe, but from 2006 onwards the virus emerged for the first time in north-western Europe, including the Netherlands, Belgium, Germany, France and the United Kingdom [6], re-emerging in France in 2015 [37]. Windborne spread of infected midges and the movement of animals, legally or otherwise, were identified as the main risks for the introduction of BTV to Ireland [35]. Given that several competent *Culicoides* arbovirus vector species are abundant in Ireland and the high density of ruminant species, it was considered that the potential for an epidemic existed should introduction occur. The application of movement controls, allied with favourable prevailing winds has resulted in Ireland remaining free of infection as demonstrated by the negative outcomes of active and passive surveillance of farmed ruminants and vector monitoring [38].

Compared to the extensive knowledge of the prevalence of antibodies to BVDV, BoHV-1, SBV and BTV in farmed ruminant species, there is a paucity of comparable data for wild deer. The purpose of this study was therefore to conduct a serological survey of free-ranging deer in ROI and NI for the presence of antibodies to these four viruses.

## Methods

### Collection and submission of samples

Two panels of blood samples, were assembled from free-ranging deer; in ROI, by licenced hunters; and in NI by staff of the Forestry Service of the Department of Agriculture, Environment and Rural Affairs.

All samplings followed a similar protocol, with hunters being provided with sampling kits, instructions, a submission form and packaging for returning samples. Briefly, the sampling kit contained a plain vacutainer tube and a sterile plastic pipette (Panel 1) or a sterile plastic universal container (Panel 2). Samples were collected post mortem from the chest cavity (Panel 1) or the inferior vena cava by incision (Panel 2).

Hunters were requested to return the submission form, providing details including the date and location of sampling, the species of deer and its sex and age (adult, yearling or calf), along with the sample material, in the packaging provided. Hunters then arranged dispatch of samples to the Central Veterinary Research Laboratory (CVRL) of the Veterinary Laboratory Service of the Department of Agriculture, Food and the Marine (DAFM).

### Serological testing

Sera were tested for antibodies to BVDV, BoHV-1, SBV and BTV using the following commercially available competitive or blocking enzyme linked immunosorbent assay (ELISA) kits according to the manufacturers’ instructions.

BVDV: SVANOVIR p80 Ab ELISA (Boehringer Ingelheim Svanova, Uppsala, Sweden). Sera with a percentage inhibition (%INH) of <45% were classified as negative and those with %INH ≥45% as positive.

BoHV-1: Herdchek IBRgB Infectious Bovine Rhinotracheitis Virus (BHV-1) gB Antibody Test Kit (IDEXX Europe B.V., The Netherlands). Sera with a percentage blocking (PB%) of <45% were classified as negative, those with PB% ≥55% as positive and those with 45% ≤ PB% <55% as inconclusive.

SBV: ID Screen Schmallenberg virus Competition Multispecies ELISA; (IDvet; Grabels, France). Sera with a sample to negative percentage (S/N%) of ≤40% were classified as positive, those with S/N% >50% as negative and those with 40% < S/N% ≤50% as inconclusive.

BTV: Bluetongue Virus Antibody Test Kit, cELISA (VMRD, Pullman, USA). Sera with a S/N% of <50% were classified as positive and those with values of ≥50% as negative.

Values for the specificity (Sp) and sensitivity (Se) of each test were taken from the published literature as follows: 98.2% and 91.6% (BVD [39]); 99.7% and 99.0% (BoHV-1 [40, 41]); 99.8% and 97.2% (SBV [42]); 99.3% and 98.6% (BTV [43]).

## Results

### Details of deer sampled

#### Panel 1

**Fig. 1:**
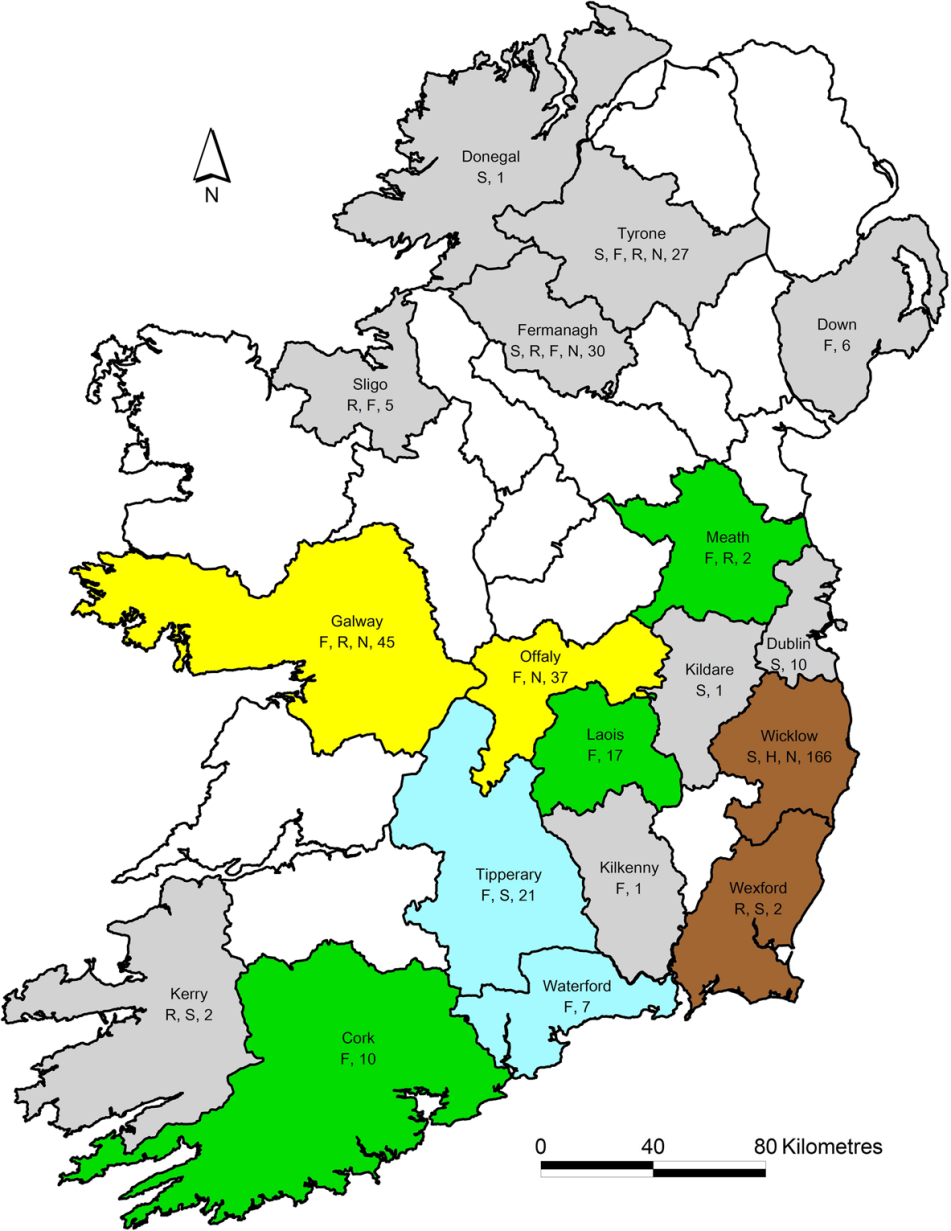
Map showing counties from which deer were submitted, including species and total number sampled in each county (F, fallow; H, Hybrid; R, Red; S, Sika; N, not recorded). Counties in which one or more positive results were recorded for SBV only, for BoHV-1 only, for both BoHV-1 and BVD and for BoHV-1, BVD and SBV are coloured *green*, *blue* and *brown*, respectively; results for the remaining counties from which samples were submitted (coloured grey) were negative for all four pathogens

Between January 2013 and March 2015, samples were submitted from a total of 259 deer (202 from ROI and 57 from NI), of which 35 (14%) were received in 2013, 205 (79%) in 2014 and 19 (7%) in 2015. These samples were collected in 18 counties (Fig. 1), reflecting published reports of the natural distribution of the deer species’ ranges [2, 44–46]. The numbers sampled per county ranged from one to forty five. Fallow deer were sampled most frequently (53%), followed by sika (35%). Further details of the numbers of deer sampled per county, and their species, is provided in Table 1. No age group details were provided for 87 deer. The majority of the remaining 172 deer were adults, with calves being sampled least frequently (Table 2). The sex of 5 deer was not provided. One hundred and forty one (56%) of the remaining 254 deer were male (Table 2).

**Table 1:** Details of the numbers (%) and species of deer sampled and the counties from which they were collected for Panel 1(top), Panel 2 (bottom) and overall

County	Fallow	Hybrid	Red	Sika	Not recorded	Total
Cork	10	-		-	-	10
Donegal	-	-		1	-	1
Down	6	-		-	-	6
Dublin	-	-		2		2
Fermanagh	1	-	7	21	1	30
Galway	39	-	3	-	3	45
Kerry		-	1	1	-	2
Kildare		-		1	-	1
Kilkenny	1	-		-	-	1
Laois	17	-		-	-	17
Meath	1	-	1		-	2
Offaly	33	-		-	4	37
Sligo	2	-	3		-	5
Tipperary	20	-		1		21
Tyrone	1	-	1	24	1	27
Waterford	7	-	-	-	-	7
Wexford	-	-	1	1		2
Wicklow	-	-	-	38	5	43
*Panel 1 total*	*138 (53.3%)*	*0* *(0%)*	*17 (6.6%)*	*90 (34.7%)*	*14* *(5.4%)*	*259 (100%)*
Wicklow	-	1	-	115	7	123
Dublin	-	-	-	8	-	8
*Panel 2 total*	*-* *(0%)*	*1* *(0.8%)*	*-* *(0%)*	*123* *(93.9%)*	*7* *(5.3%)*	*131* *(100%)*
Overall total	138 *(35.3%)*	1 *(0.3%)*	17 *(4.3%)*	213 *(54.8%)*	21 *(5.3%)*	390 *(100%)*

**Table 2:** Details of the sex and age groups of deer sampled for Panel 1 (top), Panel 2 (bottom) and overall

		Age group			
Sex	Adult	Yearling	Calf	Not recorded	Overall Total
Male	59	36	8	38	141
Female	42	13	14	44	113
Not recorded	-	-	-	5	9
Total, Panel 1	101	49	22	87	259
Male	45	12	3	5	65
Female	49	7	5	4	65
Not recorded	1	-	-	-	1
Total, Panel 2	95	19	8	9	131
Male	104	48	11	43	206
Female	91	20	19	48	178
Not recorded	1	-	-	5	6
Total Overall	196	68	30	96	390

#### Panel 2

A total of 131 samples collected between September 2014 and May 2015 were available from the archive for testing, of which 88 (67%) were collected in 2014 and 43 in 2015 (33%). With the exception of 8 samples collected in County Dublin, all samples were collected at locations in County Wicklow (Table 1). In contrast to Panel 1, sika were sampled most frequently (94%; Table 1) reflecting this species’ natural distribution within this area [2, 45, 47]. No age group was recorded for 9 deer. The majority of the remaining 122 deer were adults, with calves again being sampled least frequently (Table 2). The sex of 1 deer was not provided, with the remaining 130 deer comprising equal numbers of males and females (Table 2).

### Serological testing

Not all samples were available for testing in all assays, typically due to small volumes of sera having been collected. A total of 1 and 2 deer were not tested for BoHV-1 and SBV, respectively (Table 3). Overall 48 deer gave a positive result in one or more assays, with a total of 51 positive results recorded across all assays. Further details are provided below and in Table 4 and Fig 1. The true prevalence (Tp) and 95% confidence limits for each assay were determined using the Rogan-Gladen estimator as implemented in the Survey Toolbox of EpiTools [48].

**Table 3:** Details of the numbers of deer tested by age group for antibodies to each virus, the number testing positive and the derived true prevalence (Tp) 95% confidence intervals

	BVDV		BoHV-1		SBV		BTV	
Age Group	Tested	Positive	Tested	Positive	Tested	Positive	Tested	Positive
Adult	196	4	195	1	196	30^b^	196	0
Yearling	68	1	68	0	67	1	68	0
Calf	30	0	30	2	29	3	30	0
Not recorded	96	1	96	4^a^	96	4^c^	96	0
Total	390	6 (1.5%)	389	7 (1.8%)	388	38 (9.7%)	390	0 (0.0%)
Tp (95% CI)	0.0% (0-1.7%)		1.5% (0.1-3.0%)		9.9% (6.8-13.0%)		0.0% (0.01-0.10%)	

^a^A further 5 samples gave an inconclusive result

^b^A further 6 samples gave an inconclusive result

^c^A further 1 sample gave an inconclusive result

**Table 4:** Details of the 48 deer from which 51 positive results were recorded. Data are presented by County and date of receipt

						BoHV-1		SBV		BVDV	
Sample	Date of receipt	County	Species	Sex	Age	Value (PB%)	Result	Value (S/N%)	Result	Value (%INH)	Result
1	19/11/2014	Cork	Fallow	F	Adult	-	Neg	27.0	POS	-	Neg
2	19/11/2014	Cork	Fallow	F	NR^1^	-	Neg	12.6	POS	-	Neg
3	16/12/2014	Cork	Fallow	F	Adult	-	Neg	20.9	POS	-	Neg
4	23/01/2015	Cork	Fallow	F	NR	-	Neg	5.6	POS	-	Neg
5	08/01/2013	Galway	Fallow	F	NR	60.9	POS	-	Neg	-	Neg
6	08/01/2013	Galway	Fallow	F	NR	62.2	POS	-	Neg	-	Neg
7	08/01/2013	Galway	Fallow	F	NR	59.7	POS	-	Neg	-	Neg
8	29/10/2014	Galway	Fallow	M	Adult	-	Neg	-	Neg	75	POS
9	06/11/2014	Galway	Fallow	M	Yearling	-	Neg	-	Neg	69	POS
10	03/03/2014	Laois	Fallow	F	Adult	-	Neg	24.7	POS	-	Neg
11	16/10/2014	Meath	Red	M	Adult	-	Neg	16.0	POS	-	Neg
12	29/10/2014	Offaly	Fallow	M	NR	69.5	POS	-	Neg	95	POS
13	04/03/2014	Tipperary	Fallow	F	Adult	-	Neg	7.8	POS	-	Neg
14	10/11/2014	Tipperary	Fallow	M	Adult	-	Neg	8.4	POS	-	Neg
15	10/11/2014	Tipperary	Fallow	M	Calf	-	Neg	6.5	POS	-	Neg
16	18/11/2014	Tipperary	Fallow	M	Adult	-	Neg	11.3	POS	-	Neg
17	03/11/2014	Waterford	Fallow	M	Adult	-	Neg	14.8	POS	-	Neg
18	25/11/2014	Waterford	Fallow	F	Adult	-	Neg	19.9	POS	-	Neg
19	26/11/2014	Wexford	Red	F	Adult	98.2	POS	23.2	POS	59	POS
20	20/02/2014	Wicklow	Sika	M	Calf	98.0	POS	-	Neg	-	Neg
21	26/02/2014	Wicklow	Sika	F	Adult	-	Neg	23.0	POS	-	Neg
22	29/09/2014	Wicklow	Sika	M	Adult	-	Neg	10.1	POS	-	Neg
23	23/10/2014	Wicklow	Sika	M	Adult	-	Neg	4.5	POS	-	Neg
24	11/11/2014	Wicklow	Sika	F	Calf	-	Neg	11.5	POS	-	Neg
25	22/12/2014	Wicklow	Sika	F	Adult	-	Neg	4.3	POS	-	Neg
26	13/01/2015	Wicklow	Sika	F	Adult	-	Neg	9.8	POS	-	Neg
27	14/01/2015	Wicklow	Sika	F	NR	-	Neg	25.9	POS	-	Neg
	(continued)					BoHV-1		SBV		BVDV	
	Date of receipt	County	Species	Sex	Age	Value	Result	Value	Result	Value	Result
28	25/02/2015	Wicklow	NR	F	Adult	-	Neg	-	Neg	80	POS
29	25/02/2015	Wicklow	NR	M	Calf	55.3	POS	-	Neg	-	Neg
30	01/10/2014	Wicklow^2^	Sika	M	Adult	-	Neg	6.2	POS	-	Neg
31	28/10/2014	Wicklow^2^	Sika	F	Adult	-	Neg	5.9	POS	-	Neg
32	29/10/2014	Wicklow^2^	Sika	F	Adult	-	Neg	6.2	POS	-	Neg
33	29/10/2014	Wicklow^2^	Sika	F	Adult	-	Neg	4.2	POS	-	Neg
34	29/10/2014	Wicklow^2^	Sika	F	Calf	-	Neg	5.1	POS	-	Neg
35	07/11/2014	Wicklow^2^	Sika	F	Adult	-	Neg	7.1	POS	-	Neg
36	13/11/2014	Wicklow^2^	Sika	M	Adult	-	Neg	24.0	POS	-	Neg
37	25/11/2014	Wicklow^2^	Sika	M	Adult	-	Neg	6.8	POS	-	Neg
38	05/12/2014	Wicklow^2^	Sika	F	Yearling	-	Neg	39.6	POS	-	Neg
39	23/12/2014	Wicklow^2^	Sika	F	NR	-	Neg	5.1	POS	-	Neg
40	13/01/2015	Wicklow^2^	Sika	F	Adult	-	Neg	6.2	POS	-	Neg
41	31/03/2015	Wicklow^2^	Sika	F	Adult	-	Neg	5.5	POS	-	Neg
42	31/03/2015	Wicklow^2^	Sika	M	Adult	-	Neg	11.5	POS	-	Neg
43	31/03/2015	Wicklow^2^	Sika	F	Adult	-	Neg	19.6	POS	-	Neg
44	28/04/2015	Wicklow^2^	Sika	M	Adult	-	Neg	-	Neg	48	POS
45	28/04/2015	Wicklow^2^	Sika	F	Adult	-	Neg	6.4	POS	-	Neg
46	28/04/2015	Wicklow^2^	Sika	F	Adult	-	Neg	5.5	POS	-	Neg
47	28/04/2015	Wicklow^2^	Sika	M	Adult	-	Neg	10.0	POS	-	Neg
48	01/05/2015	Wicklow^2^	Sika	M	Adult	-	Neg	9.7	POS	-	Neg

Wicklow^2^: samples from Panel 2. Otherwise all deer were from Panel 1

^1^Not recorded

Test values are presented for positive (POS) samples only

#### BVDV

Six samples (1.5%), tested positive for BVDV antibody (Tp 0.0%, 0-1.7% CI), of which 4 were adults, one was a yearling and one for which an age group was not provided (Table 3), with test values (%INH) ranging from 48–80%. Samples that tested positive were collected from fallow and red deer in five counties, with four being collected in 2014 and two in 2015 (Table 4).

#### BoHV-1

Seven sera (1.8%), all from Panel 1, gave positive antibody results for BoHV-1 (Tp 1.5%, 0.1-3.0% CI) with test values (PB%) ranging from 55.3% to 98.2%, while a further five (1.3%) gave inconclusive results (Table 3). Eight of the twelve positive and inconclusive samples came from fallow deer in County Galway and all were received on 08.01.2013. The remaining four samples came from counties Wicklow, Offaly and Wexford and were collected in 2014 (*n* = 3) or 2015 (*n* = 1; Table 4).

#### SBV

Thirty eight sera (9.7%) collected in 2014 and 2015 gave positive antibody results for SBV (Tp 9.9%, 6.8-13.0% CI) with values ranging from to 4.2% to 39.6%, while a further eight (2.0%) gave inconclusive results, (Tables 3 and 4). Positive results were predominantly obtained from adults, although some calves were also positive. The majority of samples with positive or inconclusive results came from deer in southern and eastern counties of the ROI, including 25 from county Wicklow. However one deer sampled in Fermanagh in the western part of NI was also positive. The first positive result came from a sample from Wicklow received on 26.2.2014. Positive results were obtained from both sexes and from fallow, sika and red deer.

#### BTV

All sera tested negative for BT antibody (Tp 0.0%, 0.01-0.10% CI).

#### Co-exposures

One fallow deer sampled in County Offaly in 2014 tested positive for both BoHV-1 and BVDV, while one of the red deer sampled in Wexford in 2014 was positive for BVDV, BoHV-1 and SBV.

## Discussion

This study provides the first published information on the prevalence and distribution of a number of existing and emerging pathogens in free-ranging deer in Ireland and provides initial indications of the potential involvement they may have in the epidemiology of these pathogens. In common with a number of prevalence studies in wild deer conducted elsewhere [44, 46, 48–50], the current study is based on convenience samples provided by hunters rather than using a formal stratified sampling frame. To date there has not been a formal census of any deer species on the island of Ireland. However based on the most recent data available on the geographic distribution of the species tested [44–47], the authors consider that the results reflect the national position. As such, they provide valuable baseline data that have not previously been available and against which the results of future surveys can be benchmarked.

Antibodies to BVDV were detected in only 6 (1.5%) of samples, with these coming from fallow, red and sika deer sampled in four different counties (Galway, Offaly, Wexford and Wicklow; Tables 3 and 4). This is in contrast to the levels of seroprevalence that have been reported in farmed ruminants in Ireland. O’Neill and others [51] reported annual seroprevalence figures in juvenile and adult cattle for the years 2005–2008 ranging from 47–52% and 66–71%, respectively. Studies in adult sheep in ROI [17] and NI [16] reported seroprevalence figures of 5.6% and 5.3% respectively. The results of the current study do not provide evidence of sylvatic circulation of BVDV in deer in Ireland. The low seroprevalence detected is consistent with deer being a spillover host for BVDV, most likely from the cattle population where the highest level of exposure has historically been found, rather than a reservoir host from which infection could be reintroduced. This is particularly important in light of the progress being made toward eradication of BVDV from the cattle population, where the prevalence of PI births has decreased from 0.66% in 2013 to 0.14% in mid-2016 [52].

A number of other European countries have also investigated the seroprevalence of BVDV in deer, typically to examine their epidemiological importance in the context of national eradication programmes. A sero-survey using a virus neutralisation (VN) test of free-living deer from regions of Denmark with a relatively high prevalence of cattle herds with a persistent BVD infection status prior to its eradication from cattle found a very low prevalence of cervid infection, with only 3 positives detected in 476 tested [53]. The authors concluded that the positive animals were likely to have resulted from transmission from cattle to deer, that transmission among deer or from deer to cattle was highly unlikely and therefore that the possibility of free-ranging deer being a source of infection for cattle was remote.

A serological survey of samples collected in Norway between 1993 and 2000 found 78 of 635 (12.3%) roe deer to be seropositive to BVDV by ELISA or VN testing, with the authors concluding that pestivirus is endemic in this species [28]. While the authors at that time noted the possibility of deer to cattle transmission impacting on eradication and surveillance within the Norwegian eradication, this risk has proven unfounded as demonstrated by the successful completion of the eradication programme [49].

The role of wild ruminants, including red and roe deer, in the epidemiology of BVDV infections in domestic livestock in Switzerland was investigated [50]; the authors found that despite regular interactions with farmed ruminants, infection in wild ruminants was sporadic with virus neutralising (VN) antibodies not found in any of 435 roe deer and detected in 13/476 red deer (2.7%). They concluded that wildlife was an incidental spillover host rather than a reservoir host for BVDV and as such did not represent a threat to the Swiss National BVDV eradication programme in livestock [50].

A recent study in Belgium [54] screened wild roe deer in Flanders for serological evidence of exposure to a range of pathogens, including BVDV. Despite an expanding population and regular contact with livestock, only 1.3% were found seropositive, leading to the conclusion that they do not play an important role in the epidemiology of infection in domestic animals.

More recently, a similar pestivirus study was conducted in the south of Spain [55], where wild ruminant populations have also increased substantially, resulting in the frequent sharing of habitats with domestic livestock. This found only 1 of 892 red deer to be seropositive and concluded that the deer were spillover hosts only and did not represent a risk for domestic ruminants. Another study of sympatric alpine populations of livestock and wild ruminants, including deer in north-west Spain generated similar findings [56].

The susceptibility of free-ranging red, roe and fallow deer to infection with BoHV-1 has previously been demonstrated [24]. However, an extensive serological cross-reactivity between ruminant alphaherpesviruses, including CvHV-1 and -2, has been demonstrated by both VN testing and blocking (gB) ELISA [25, 28, 57]. As a result it is not possible to confirm the identity of the alphaherpes virus species generating the antibodies detected with the BoHV-1 gB ELISA in the current study without further cross-neutralisation studies [58]. Given that the natural host of CvHV-2 is considered to be reindeer (*Rangifer tarandus*), it would seem likely that the positive results are most likely attributable to either BoHV-1 or to CvHV-1. While infection with CvHV-1 is considered to be widespread in Europe [27], particularly in red deer, further studies are required to confirm its presence in Ireland.

Irrespective of the alphaherpes virus species present in deer in Ireland, their potential to confound BoHV-1 eradication programmes in cattle has been suggested [23, 27, 28]. This could arise either through deer acting as a reservoir host for BoHV-1 from which reintroduction to cattle could occur, initiating new outbreaks, or as a source from which CvHV-1 or -2 could be transmitted, resulting in false positive serological results for BoHV-1. In practice however, the results of an experimental study in red deer showing no transmission to in contact deer caused the authors to conclude that eradication of BoHV-1 in deer, if present, was not required for successful eradication in the cattle population [23]. Conversely, the presence of other alphaherpesviruses in deer has not prevented the successful eradication of BoHV-1 in cattle in Norway and Sweden [59, 60].

Taken together with the low prevalence of antibodies found in the current study, this suggests firstly that deer are not a reservoir host of BoHV-1 for cattle and that secondly the current low prevalence of alphaherpesvirus infection detected does not pose a major threat to bovine IBR control at herd, regional or national level in Ireland. However, the observation that 8 of the 12 samples that tested positive or inconclusive for BoHV-1 were received in a single submission from fallow deer in Galway indicates that the prevalence of infection may be higher in some deer populations. Depending on the virus to which the antibodies were produced and the degree of contact between this population of deer and cattle in the vicinity, this may indicate that the possibility of transmission between these two species cannot be excluded.

The highest seroprevalence (9.7%) detected in the current study was to SBV, with 38 of 388 deer testing positive (Table 3). The majority of positive samples came from adult deer sampled in counties in the south and east of Ireland, particularly county Wicklow, where 25 of 174 (Tables 1 and 4) deer tested positive. While the largest number of positive results were reported from deer in Wicklow, the underlying variation in the number of samples submitted per county must also be borne in mind (Table 1). Thus 25 of 166 deer from Wicklow tested positive, with lower numbers of positive SBV results reported from other counties. However, the proportion testing positive was higher in several of these counties e.g. 4 of 10 from Cork, 2 of 7 from Waterford, 1 of 2 from Wexford (Tables 1 and 4). The variation in submission levels per county is considered to reflect variation between counties in the level of hunting and the degree of engagement of the hunters with the survey. This geographical distribution is consistent with the results of serological surveys of both cattle [34] and sheep [33] in Ireland. The relatively low seroprevalence among younger deer is consistent with failure of this virus to circulate openly beyond 2012. Serological surveillance studies conducted in Irish cattle from 2013–2015 likewise reported a very low seroprevalence in young stock [61], providing further evidence for the absence of continued circulation of SBV. Seroprevalence studies for SBV in wild cervids have been conducted elsewhere in Europe. One study reported an average seroprevalence in red deer of 20% in 9 departments in France during 2011–12 [62]. In Belgium, a seroprevalence of 43.1% was reported in red and roe deer sampled in Wallonia in the autumn of 2011 [63], while in Flanders a 63% seroprevalence was reported in roe deer sampled in 2012 [54]. These higher seroprevalence levels relative to those reported in the current study may reflect differences between studies in the timing of sampling relative to the emergence of SBV, the failure of SBV to become established in Ireland [61] or underlying ecological or epidemiological differences.

Only two samples provided evidence of co-exposure, one for BoHV-1 and BVDV and one for BoHV-1, BVD and SBV. Overall this low frequency of co-exposure is consistent with the low prevalences recorded for each of these three pathogens, particularly BVDV and BoHV-1.

The failure to detect any antibodies to BTV in deer in this study is consistent with the island of Ireland retaining freedom from this virus [38]. However, the presence of suitable BTV vector species in Ireland [6, 61] indicates that the virus could become established if introduced and highlights the need for continued surveillance.

## Conclusion

The current study reports the findings of the first serological survey of wild deer in Ireland for a range of viral pathogens. The results are consistent with a very low seroprevalence for both BVD and alphaherpes viruses. In both cases the results suggest that the presence of these viruses in deer is not a significant risk to their control and eradication from the cattle population. While deer can become infected with BVDV as a spillover host from cattle, further work is required to characterise the alphaherpes virus generating antibodies detectable using the BoHV-1 gB ELISA. The SBV results show consistency with those reported from cattle and sheep, suggesting that the distribution in these species provides a reliable indication of the distribution in deer also. Overall, the results provide a baseline against which future surveys of either wild or farmed/captive populations of deer can be compared. While the focus of the current work was on viral pathogens, the approach taken could also be readily expanded to consider bacterial pathogens and parasites.

## Abbreviations

%INH: % inhibition
BoHV-1: bovine herpesvirus-1
BTV: bluetongue virus
BVDV: bovine viral diarrhoea virus
CvHV-1: cervid herpesvirus-1
CvHV-2: cervid herpesvirus-2
ELISA: enzyme linked immunosorbent assay
IBR: infectious bovine rhinotracheitis
NI: Northern Ireland
PB%: percentage blocking
ROI: Republic of Ireland
S/N%: sample to negative percentage
SBV: Schmallenberg virus
VN: virus neutralising

## References

[1] Carden RF, McDevitt AD, Zachos FE, Woodman PC, O’Toole P, Rose H, Monaghan NT, Campana MG, Bradley DG, Edwards CJ (2012). Phylogeographic, ancient DNA, fossil and morphometric analyses reveal ancient and modern introductions of a large mammal: The complex case of red deer (*Cervus elaphus*) in Ireland. Quat Sci Rev.

[2] Carden RF, Carlin CM, Marnell F, McElholm D, Hetherington J, Gammell MP (2011). Distribution and range expansion of deer in Ireland. Mamm Rev.

[3] National Biodiversity Data Centre. http://www.biodiversityireland.ie/projects/invasive-species/species-alerts/alert-tracking-table/ Accessed 5 Dec 2016.

[4] Rourke EO, Flynn CO. Risk Assessment of *Capreolus capreolus* 2014. p. 1–24. http://nonnativespecies.ie/wp-content/uploads/2014/03/Capreolus-capreolus-Roe-Deer.pdf. Accessed 5 Dec 2016.

[5] Wernike K, Conraths F, Zanella G, Granzow H, Gache K, Schirrmeier H (2014). Schmallenberg virus-Two years of experiences. Prev Vet Med.

[6] Wilson AJ, Mellor PS (2009). Bluetongue in Europe: past, present and future. Philos Trans R Soc Lond B Biol Sci.

[7] Graham D, Lynch M, Coughlan S, Doherty ML, O’Neill R, Sammin D (2014). Development and review of the voluntary phase of a national BVD eradication programme in Ireland. Vet Rec.

[8] Graham DA, Clegg TA, O’Sullivan P, More SJ (2015). Influence of the retention of PI calves identified in 2012 during the voluntary phase of the Irish national bovine viral diarrhoea virus (BVDV) eradication programme on herd-level outcomes in 2013. Prev Vet Med.

[9] Lanyon S, Hill F, Reichel M, Brownlie J (2014). Bovine viral diarrhoea: Pathogenesis and diagnosis. Vet J.

[10] Lindberg AL, Alenius S (1999). Principles for eradication of bovine viral diarrhoea virus (BVDV) infections in cattle populations. Vet Microbiol.

[11] Cowley DJB, Clegg TA, Doherty ML, More SJ (2012). Bovine viral diarrhoea virus seroprevalence and vaccination usage in dairy and beef herds in the Republic of Ireland. Ir Vet J.

[12] Cowley DJB, Graham DA, Guelbenzu M, Doherty ML, More SJ (2014). Aspects of bovine herpesvirus 1 and bovine viral diarrhoea virus herd-level seroprevalence and vaccination in dairy and beef herds in Northern Ireland. Ir Vet J.

[13] Passler T, Walz PH (2010). Bovine viral diarrhea virus infections in heterologous species. Anim Health Res Rev.

[14] Passler T, Walz P, Ditchkokk S, Brock K, Deyoung R, Foley A (2009). Cohabitation of pregnant white-tailed deer and cattle persistently infected with Bovine viral diarrhea virus results in persistently infected fawns. Vet Microbiol.

[15] Passler T, Walz PH, Ditchkoff SS, Givens MD, Maxwell HS, Brock KV (2007). Experimental persistent infection with bovine viral diarrhea virus in white-tailed deer. Vet Microbiol.

[16] Graham DA, Calvert V, German A, Mccullough SJ (2001). Pestiviral infections in sheep and pigs in Northern Ireland. Vet Rec.

[17] O’Neill RG, O’Connor M, O’Reilly PJ (2004). A survey of antibodies to pestivirus in sheep in the Republic of Ireland. Ir Vet J.

[18] Infectious bovine rhinotracheitis/infectious pustular vulvovaginitis. In: Man Diagnostic Tests Vaccines Terr. Anim. 2016. p. Chapter 2.14.12. http://www.oie.int/fileadmin/Home/eng/Health_standards/tahm/2.04.12_IBR_IPV.pdf. Accessed 22 Oct 2016.

[19] Nandi S, Kumar M, Manohar M, Chauhan RS (2009). Bovine herpes virus infections in cattle. Anim Health Res Rev.

[20] Cowley DJB, Clegg TA, Doherty ML, More SJ (2011). Aspects of bovine herpesvirus-1 infection in dairy and beef herds in the Republic of Ireland. Acta Vet Scand.

[21] Graham DA (2013). Bovine herpes virus-1 (BoHV-1) in cattle-a review with emphasis on reproductive impacts and the emergence of infection in Ireland and the United Kingdom. Ir Vet J.

[22] Chow TL, Davis RW (1964). The susceptibility of mule deer to infectious bovine rhinotracheitis. Am J Vet Res.

[23] Mollema L, Rijsewijk FAM, Nodelijk G, De Jong MCM (2005). Quantification of the transmission of bovine herpesvirus 1 among red deer (*Cervus elaph*us) under experimental conditions. Vet Microbiol.

[24] Kálmán D, Egyed L (2005). PCR detection of bovine herpesviruses from nonbovine ruminants in Hungary. J Wildl Dis.

[25] Thiry J, Keuser V, Muylkens B, Meurens F, Gogev S, Vanderplasschen A (2006). Ruminant alphaherpesviruses related to bovine herpesvirus 1. Vet Res.

[26] Squires R, Wilson P, Whelan N, Johnstone A, Ayanegui-Alcérreca M, Castillo-Alcala F (2012). Alpha and gamma herpesvirus detection in two herds of farmed red deer (*Cervus elaphus*) in New Zealand. N Z Vet J.

[27] Thiry J, Widén F, Grégoire F, Linden A, Belák S, Thiry E (2007). Isolation and characterisation of a ruminant alphaherpesvirus closely related to bovine herpesvirus 1 in a free-ranging red deer. BMC Vet Res.

[28] Lillehaug A, Vikøren T, Larsen I-L, Akerstedt J, Tharaldsen J, Handeland K (2003). Antibodies to ruminant alpha-herpesviruses and pestiviruses in Norwegian cervids. J Wildl Dis.

[29] Bradshaw B, Gaynor S. Evidence that exposure to Schmallenberg virus has been quite widespread in Southern and South-eastern counties of Ireland during 2012. http://www.agriculture.gov.ie/media/migration/animalhealthwelfare/diseasecontrols/schmallenbergvirus/Schmallenbergvirussummary17Jan13.pdf. 2013. Accessed 09 May 2017

[30] All-island Animal Disease Surveillance Report 2012 A joint AFBI / DAFM Veterinary Laboratories publication. http://www.agriculture.gov.ie/media/migration/animalhealthwelfare/labservice/rvlreports/All%20Island%20Animal%20Disease%20Surveillance%20Report%202012.pdf. Accessed 09 May 2017.

[31] Johnson A, Bradshaw B, Boland C, Ross P (2014). A bulk milk tank study to detect evidence of spread of Schmallenberg virus infection in the south-west of Ireland in 2013. Ir Vet J.

[32] All-island Animal Disease Surveillance Report 2013 A joint AFBI / DAFM Veterinary Laboratories publication. http://www.agriculture.gov.ie/media/migration/animalhealthwelfare/labservice/rvlreports/2013%20AFBI-DAFM%20All-island%20Surveillance%20Report.pdf. Accessed 09 May 2017.

[33] Barrett DJ, More SJ, O’Neill RG, Collins DM, O’Keefe C, Regazzoli V, Sammin D (2015). Exposure to Schmallenberg virus in Irish sheep in 2013. Vet Rec.

[34] Barrett D, More SJ, O’Neill R, Bradshaw B, Casey M, Keane M, Sammin D (2015). Prevalence and distribution of exposure to Schmallenberg virus in Irish cattle during October 2012 to November 2013. BMC Vet Res.

[35] Veterinary Risk Analysis : Introduction of Bluetongue Virus into Ireland from Bluetongue Restricted Areas in other Member States. 2008. p. 1–25. http://www.agriculture.gov.ie/media/migration/animalhealthwelfare/diseasecontrols/bluetonguedisease/Bluetongue_RA_Final_150508.pdf. Accessed 5 Dec 2016.

[36] Menzies FD, McCullough SJ, McKeown IM, Forster JL, Jess S, Batten C (2008). Evidence for transplacental and contact transmission of bluetongue virus in cattle. Vet Rec.

[37] Bréard E, Sailleau C, Quenault H, Lucas P, Viarouge C, Touzain F (2016). Complete Genome Sequence of Bluetongue Virus Serotype 8, Which Reemerged in France in August 2015. Genome Announc.

[38] Bluetongue. http://nahsp.agriculture.gov.ie/diseases/individualdiseaselistings/bluetongue/ Accessed 5 Dec 2016.

[39] Xia H, Liu L, Nordengrahn A, Kiss I, Merza M, Eriksson R (2010). A microsphere-based immunoassay for rapid and sensitive detection of bovine viral diarrhoea virus antibodies. J Virol Methods.

[40] Kramps JA, Magdalena J, Quak J, Weerdmeester K, Kaashoek MJ, Veldhuis MAM (1994). A Simple, Specific, and Highly Sensitive Blocking Enzyme-Linked Immunosorbent Assay for Detection of Antibodies to Bovine Herpesvirus 1. J Clin Microbiol.

[41] de Wit JJ, Hage JJ, Brinkhof J, Westenbrink F (1998). A comparative study of serological tests for use in the bovine herpesvirus 1 eradication programme in The Netherlands. Vet Microbiol.

[42] Balmer S, Gobet H, Nenniger C, Hadorn D, Schwermer H, Vögtlin A (2015). Schmallenberg virus activity in cattle in Switzerland in 2013. Vet Rec.

[43] Niedbalski W (2012). Evaluation of commercial ELISA kits for the detection of antibodies against bluetongue virus. Pol J Vet Sci.

[44] Carden R, Lysaght L, Marnell F (2016). Fallow deer (*Dama dama*). Atlas Mamm. Irel. 2010- 2015.

[45] Carden R, Lysaght F, Marnell L (2016). Sika deer (*Cervus nippon*). Atlas Mamm. Irel. 2010-2015.

[46] Burkitt T, Lysraght L, Marnell F (2016). Red deer (*Cervus elaphus*). Atlas Mamm. Irel. 2010- 2015.

[47] Smith SL, Carden RF, Coad B, Birkitt T, Pemberton JM (2014). A survey of the hybridisation status of Cervus deer species on the island of Ireland. Conserv Genet.

[48] EpiTools. https://www.ausvet.com.au/about/resources. Accessed 18 Apr 2017.

[49] Løken T, Nyberg O (2013). Eradication of BVDV in cattle: the Norwegian project. Vet Rec.

[50] Casaubon J, Vogt H-R, Stalder H, Hug C, Ryser-Degiorgis M-P (2012). Bovine viral diarrhea virus in free-ranging wild ruminants in Switzerland: low prevalence of infection despite regular interactions with domestic livestock. BMC Vet Res.

[51] Neill RO, Wilson B, Regan C, Connaghan E, Mooney J (2008). Patterns of infection with BVD virus in laboratory submissions. Ir Vet J.

[52] Animal Health Ireland. http://animalhealthireland.ie/?page_id=229. Accessed 5 Dec 2016.

[53] Nielsen SS, Roensholt L, Bitsch V (2000). Bovine Virus Diarrhea Virus in Free-Living Deer from Denmark. J Wildl Dis.

[54] Tavernier P, Sys SU, De Clercq K, De Leeuw I, Caij AB, De Baere M (2015). Serologic screening for 13 infectious agents in roe deer (*Capreolus capreolus*) in Flanders. Infect Ecol Epidemiol.

[55] Paniagua J, García-Bocanegra I, Arenas-Montes A, Berriatua E, Espunyes J, Carbonero A (2016). Absence of circulation of *Pestivirus* between wild and domestic ruminants in southern Spain. Vet Rec.

[56] Fernández-Aguilar X, López-Olvera JR, Marco I, Rosell R, Colom-Cadena A, Soto-Heras S, et al. Pestivirus in alpine wild ruminants and sympatric livestock from the Cantabrian Mountains, Spain. Vet Rec. 2016; doi/10.1136/vr.103577 10.1136/vr.103577 27083871

[57] Das Neves CG, Roger M, Yoccoz NG, Rimstad E, Tryland M (2009). Evaluation of three commercial bovine ELISA kits for detection of antibodies against Alphaherpesviruses in reindeer (*Rangifer tarandus tarandus*). Acta Vet Scand.

[58] Frölich K, Hamblin C, Parida S, Tuppurainen E, Schettler E (2006). Serological survey for potential disease agents of free-ranging cervids in six selected national parks from Germany. J Wildl Dis.

[59] Åkerstedt J, Norström M, Mørk T. Surveillance programmes for terrestrial and aquatic animals in Norway The surveillance and control programme for infectious bovine rhinotracheitis ( IBR ) and infectious pustular vulvovaginitis ( IPV) in Norway 2014. Oslo Nor. Vet. Institute. 2015. http://www.vetinst.no/overvaking/IBR-IPV-storfe. Accessed 20 Mar 2017.

[60] Kautto AH, Alenius S, Mossing T, Becher P, Belák S, Larska M (2012). Pestivirus and alphaherpesvirus infections in Swedish reindeer (*Rangifer tarandus tarandus* L.). Vet Microbiol.

[61] Collins ÁB, Barrett D, Doherty ML, Larska M, Mee JF (2016). Post-epidemic Schmallenberg virus circulation: parallel bovine serological and Culicoides virological surveillance studies in Ireland. BMC Vet Res.

[62] Laloy E, Bréard E, Sailleau C, Viarouge C, Desprat A, Zientara S, et al. Schmallenberg Virus Infection among Red Deer. Emerg Infect Dis. 2014; doi:10.3201/eid2001.1304114. 10.3201/eid2001.130411 PMC3884713 24377838

[63] Linden A, Desmecht D, Volpe R, Wirtgen M, Gregoire F, Pirson J, et al. Epizootic Spread of Schmallenberg Virus among Wild Cervids, Belgium, Fall 2011. Emerg Infect Dis. 2012; doi:10.3201/eid1812.121067. 10.3201/eid1812.121067 PMC3557893 23171763

